# Curcumin’s Nanomedicine Formulations for Therapeutic Application in Neurological Diseases

**DOI:** 10.3390/jcm9020430

**Published:** 2020-02-05

**Authors:** Bahare Salehi, Daniela Calina, Anca Oana Docea, Niranjan Koirala, Sushant Aryal, Domenico Lombardo, Luigi Pasqua, Yasaman Taheri, Carla Marina Salgado Castillo, Miquel Martorell, Natália Martins, Marcello Iriti, Hafiz Ansar Rasul Suleria, Javad Sharifi-Rad

**Affiliations:** 1 Student Research Committee, School of Medicine, Bam University of Medical Sciences, Bam 44340847, Iran; bahar.salehi007@gmail.com; 2 Department of Clinical Pharmacy, University of Medicine and Pharmacy of Craiova, 200349 Craiova, Romania; 3 Department of Toxicology, University of Medicine and Pharmacy of Craiova, 200349 Craiova, Romania; ancadocea@gmail.com; 4 Department of Natural Products Research, Dr. Koirala Research Institute for Biotechnology and Biodiversity, Kathmandu 44600, Nepal; koirala.biochem@gmail.com (N.K.); sushantarl23@gmail.com (S.A.); 5 Italian National Research Council, Rome (CNR), 98158 Messina, Italy; lombardo@ipcf.cnr.it; 6 Department of Environmental and Chemical Engineering, University of Calabria, 87036 Rende (CS), Italy; luigi.pasqua@unical.it; 7 Phytochemistry Research Center, Shahid Beheshti University of Medical Sciences, Tehran 1991953381, Iran; taaheri.yasaman@gmail.com; 8 Facultad de Medicina, Universidad del Azuay, 14-008 Cuenca, Ecuador; csalgado@uazuay.edu.ec; 9 Department of Nutrition and Dietetics, Faculty of Pharmacy, University of Concepcion, Concepcion 4070386, Chile; 10 Unidad de Desarrollo Tecnológico, Universidad de Concepción UDT, Concepcion 4070386, Chile; 11 Faculty of Medicine, University of Porto, Alameda Prof. HernâniMonteiro, 4200-319 Porto, Portugal; 12 Institute for Research and Innovation in Health (i3S), University of Porto, 4200-135 Porto, Portugal; 13 Department of Agricultural and Environmental Sciences, Milan State University, 20133 Milan, Italy; marcello.iriti@unimi.it; 14 Department of Agriculture and Food Systems, The University of Melbourne, Melbourne 3010, Australia; hafiz.suleria@unimelb.edu.au; 15 Zabol Medicinal Plants Research Center, Zabol University of Medical Sciences, Zabol 61615-585, Iran

**Keywords:** curcumin, nanocurcumin, neurological disorders, nanocarriers, liposomes

## Abstract

The brain is the body’s control center, so when a disease affects it, the outcomes are devastating. Alzheimer’s and Parkinson’s disease, and multiple sclerosis are brain diseases that cause a large number of human deaths worldwide. Curcumin has demonstrated beneficial effects on brain health through several mechanisms such as antioxidant, amyloid β-binding, anti-inflammatory, tau inhibition, metal chelation, neurogenesis activity, and synaptogenesis promotion. The therapeutic limitation of curcumin is its bioavailability, and to address this problem, new nanoformulations are being developed. The present review aims to summarize the general bioactivity of curcumin in neurological disorders, how functional molecules are extracted, and the different types of nanoformulations available.

## 1. Introduction

Curcumin (CUR), also known as diferuloylmethane, is a turmeric (*Curcuma longa* L. rhizomes)-derived polyphenol, with multiple applications in traditional medicine for more than 2000 years [1,2]. Its use as a food ingredient and industrial dye, in cosmetics and medicinal products formulation, and even to alleviate muscle pain and inflammation and treat various pathological conditions, such as rheumatoid arthritis, gastrointestinal and inflammatory disorders, intermittent fever, renal problems, and leukoderma are amongst to the most commonly reported applications [3]. CUR was described in 1815 by Vogel and Pelletier [4] as a mixture of resin and turmeric oil. Later, in 1842, Vogel Jr. obtained the pure form of curcumin, and 68 years later, Milobedzka and Lampe identified its structure as (1*E*,6*E*)-1,7-bis(4-hydroxy-3-methoxyphenyl)-1,6-heptadiene-3,5-dione [5]. In the past decades, CUR has received a high interest due to its anti-inflammatory, antioxidant and immunomodulatory effects [6], and its benefits in cancer [7], cardiovascular diseases [8,9], diabetes mellitus [10], autoimmune diseases [11,12], and brain or psychiatric conditions [13,14,15]. Regarding the latter, there is a particular focus on CUR impact in cognition [16], dementia, Alzheimer’s disease (AD) [17,18], schizophrenia [14], and depression [19].

Neurological disorders are a significant cause of human deaths worldwide. Based on a World Health Organization (WHO) report in 2015, near 12% of global mortality was caused by neurological disorders. Among them, AD and other dementias represent a high percentage of the total deaths compared to others, consisting of 2.84% percent of mortality in high-income countries in 2005 [20]. Since neurological disorders such as AD mostly affect elderly individuals, the worldwide aging of the population has caused an increase in the human and economic burden. So far, no treatment to cure or reverse AD has been approved, but new studies could change this picture.

Several studies have shown that polyphenols such as CUR can modulate cellular signaling pathways involved in cognitive processes, such as cAMP-response element-binding protein (CREB) signaling and brain-derived neurotrophic factor (BDNF) activation [21,22]. This is crucial for both neurons’ development and survivaland for synaptic plasticity. Polyphenols play a beneficial role in maintaining brain health [23]. They are potent antioxidants, and their presence in the diet decreases the markers of oxidative stress, which reduces the risk of neurological diseases [24]. Besides, memory, attention and concentration are enhanced by polyphenols, which may contribute to improved cerebral blood flow [25]. Adding the fact that CUR is inexpensive and has little to no side effects [26], it becomes a strong candidate for a neuroprotective agent.

However, CUR and its metabolites’ application is limited due to its weak absorption, rapid metabolism, rapid systemic elimination, limited blood-brain barrier (BBB) permeability and, the most challenging factor, a low water solubility (0.4 μg/mL at normal gastric pH: 1.5–4) [27,28]. Many different formulations have been developed to resolve these issues [29,30]. In general, these innovative mechanisms improve CUR’s bioavailability by increasing its chemical stability and solubility, better permeability, and tissue distribution [31,32]. Despite the challenges that need to be answered, CUR possesses numerous advantages such as excellent biological and pharmacological activity, extensive clinical trials, and low side effects that can lead to CUR formulations becoming medicine. This has inspired scientists to develop CUR nanomedicine formulations for better bioavailability, efficacy, and therapeutic index.

As might be expected, specific differences in these novel formulations can influence their efficacy, explaining why most of the clinical trials show conflicting results regarding the beneficial effects of CUR in brain diseases, especially when it comes to the treatment of AD [33]. For this reason, we must understand how existing CUR nanomedicine formulations act, including their particular benefits and disadvantages in AD and other brain conditions. This literature review aims to summarize the general bioactivity of CUR in neurological disorders, how functional molecules are extracted, and the different nanoformulation types available.

## 2. Chemical Properties of Curcumin

**Figure 1:**
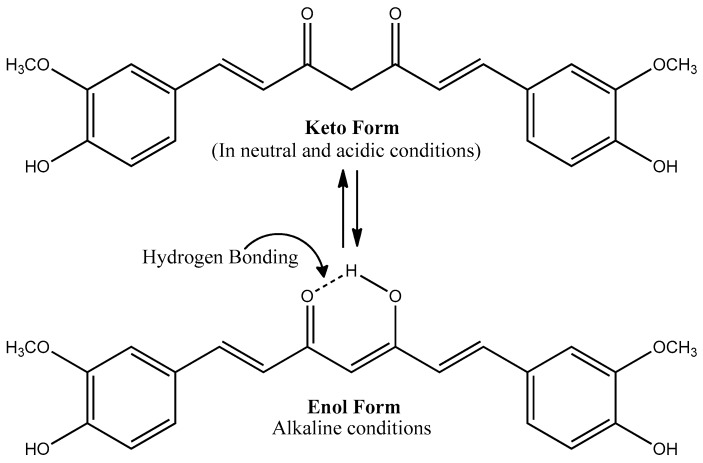
Chemical structure of curcumin and equilibrium between keto and enol tautomerism.

Chemically, CUR has two ferulic acid moieties connected by an additional carbon to shorten the carboxyl groups. It has seven carbon linkers and three major functional groups: an aromatic methoxy phenolic group; α,β-unsaturated β-diketo linker and keto-enol tautomerism [34]. CUR exists in tautomeric keto and enol conformations in equilibrium due to the intramolecular hydrogen atoms transfer at the β-diketone chain (Figure 1). The relative concentrations of keto-enol tautomers may vary depending on the temperature, pH, solvent polarity, and aromatic ring substitution [35]. In neutral and acidic aqueous solutions (from pH 3 to 7), the keto form dominates CUR, which is capable of transferring H-atom’s crucial for antioxidant activity. However, under alkaline conditions (≥pH 8), the enolic form predominates, and the phenolic part of the molecule is a major contributor for antioxidant activity through electron donation [36]. CUR’s aromatic groups provide hydrophobicity of compound, resulting in poor water solubility [37]. Typical CUR composition of commercial varieties is the combination of CUR (~77%), desmethoxycurcumin (~17%) and bisdemethoxycurcumin (~3%) known as curcuminoids [38]. 

## 3. Curcumin and Neurological Disorders

**Figure 2:**
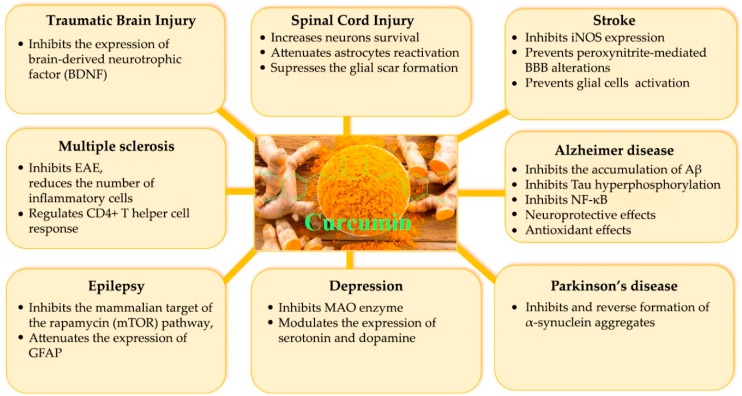
Potential mechanisms and applications of curcumin in neurological and psychiatric disorders [40,41,42,43,48,49,50,51,55,56,57]. Legend: experimental allergic encephalomyelitis (EAE), Glial fibrillary acidic protein (GFAP), inducible nitric oxide synthase (iNOS), Blood Brain Barrier (BBB).

CUR has shown promising therapeutic potential in the management of biliary disorders, LDL oxidation, blood cholesterol, anorexia, cough, diabetic wounds, thrombosis, hepatic disorders, rheumatism, sinusitis, inflammations and wounds [39]. Over the past half-decade, CUR has gained attention as a key molecule in neurological disorders, being tested both for its effectiveness and safety. Numerous preclinical studies have suggested its use in treating disorders such as AD [40,41], Parkinson disease (PD) [42,43], multiple sclerosis (MS) [44,45], migraine [46,47], epilepsy [48,49], stroke [50,51], traumatic brain injury [52,53], and spinal cord injury [54]. Various studies indicate that the antioxidant, anti-inflammatory, anti-amyloidogenic, antidepressant, antidiabetic, and antiaging properties of CUR are responsible for the neuroprotective effects as in Figure 2 [55,56,57].

At the molecular level, CUR reduces the reactive oxygen species (ROS) and advanced glycation products generation and accumulation, by down-regulating the nicotinamide adenine dinucleotide phosphate oxidaseexpression [58]. Moreover, CUR also inhibits the activation of glial cells, reduces the nuclear factor kappa B (NF-κB) activity and decrease the activity of proinflammatory interleukin (IL)-1β; IL-6, and IL-8 and cytokines (tumor necrosis factor-α (TNF-α)) in the neuronal system [44]. Furthermore, CUR attributes in metalloproteinase-9, inducible nitric oxide synthase (iNOS), cyclooxygenase-2 (COX-2), and 5-lipoxygenase (5-LOX) downregulation, where proteins related to antioxidant defense (hemeoxygenase-1 (HO-1) and heat shock proteins are upregulated to prevent the neuronal disorder [59,60].

### 3.1. Curcumin in Alzheimer’s Disease

AD is a pathological condition determined by neurofibrillary tangle (NFT) and senile plaque (SP) aggregation, severe neuroinflammation, synaptic, and neuronal loss [61]. The loss of neurons and synapses determine atrophy of the cerebral cortex, mainly in the temporal, parietal and frontal lobe. An important pathogenic event in the development of AD is the sequential proteolysis of the transmembrane amyloid-β precursor protein (AβPP) by β-APP cleaving enzyme 1 (BACE1) and γ–secretase in Aβ peptides and its aggregation around the cells. β-amyloid peptides (Aβ) deposition is the first event that triggers NFT formation, cell death, and finally, dementia [62]. Besides Aβ plaques, the other evidence for AD is the hyper-phosphorylation tau that induces disruption of microtubules and intracellular transport. However, most of the evidence suggests that inhibition of Aβ accumulation is an ideal target for pharmacotherapy [63]. 

**Figure 3:**
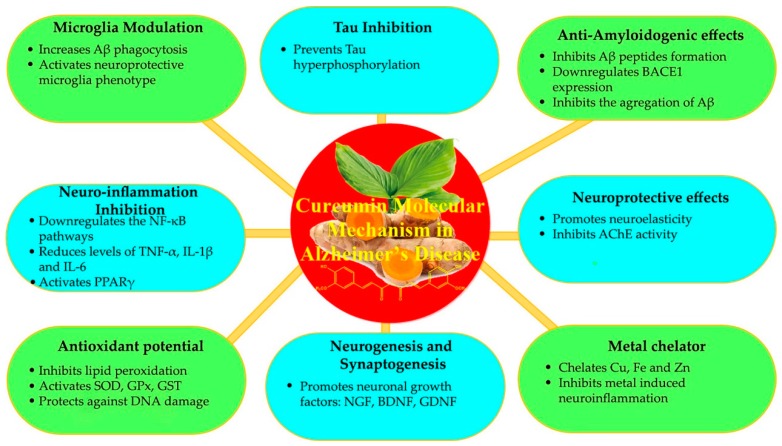
Multiple molecular mechanisms of curcumin to ameliorate Alzheimer’s disease [40,62,65,66]. Legend: β-secretase 1 (BACE1), nuclear factor κ B (NF-κB), tumour necrosis factor α (TNF α), Interleukin I beta (IL-1β), Interleukin 6 (IL-6), Peroxisome proliferator-activated receptor γ (PPAR-γ), superoxide dismutase (SOD), glutathione peroxidase (GPx), glutathione (GSH), nerve growth factor (NGF), brain-derived neurotrophic factor (BDNF), glial cell-derived neurotrophic factor (GDNF), acetylcholine esterase (AChE).

Despite the remarkable advances in the knowledge of AD pathogenesis, the typical selective neurodegeneration of the AD brains is not fully understood. Due to the lack of an early diagnosis before the onset of symptoms, approved effective disease-modifying treatments are not currently available. However, there are few clinical treatments that slow down disease progression and control symptoms [64]. Based on the old cholinergic hypothesis for the etiology AD, acetylcholinesterase (AChE) inhibitors were used to maintain the level of acetylcholine and reverse the symptoms of short-term memory loss and confusion caused by a loss in cholinergic neurons. However, none of these drugs is curative, acting mainly through reducing the degeneration of cholinergic neurons and the progression of the disease [62]. The current research focuses on the development of novel treatment, which helps to restore the degenerated neurons. CUR is a natural product extensively used in India. Indian epidemiological studies showed that the incidence of AD in this country is the lowest worldwide. The relationship between CUR consumption in India and lower AD prevalence is the basic to investigate the protective mechanisms of CUR in AD [65,66]. Multiple molecular mechanisms (Figure 3) have been scientifically verified, where CUR appears as an upcoming therapeutic candidate for AD prevention, treatment, and diagnosis [40].

#### 3.1.1. Aβ Peptides Inhibition

Evidence suggests that Aβ plaques formation and accumulation is prevented by CUR. CUR’s enol forms, which stain the amyloid plaques and neurofibrillary tangles (NFTs)in the brain demonstrate its binding to Aβ fibrils [67]. Zhang et al. [68] have developed aCUR fluorescence analogs with binding affinity to aggregated β-amyloid (CRANAND-2) or solubleβ-amyloid (CRANAND-58). These molecules have been increasingly detected in AD transgenic mice before plaques can be observed.

Multiple literature records have demonstrated that CUR inhibits the formation of Aβ peptides. Intragastric CUR administration to an AD mice model reduced Aβ formation by downregulating BACE1 expression, the enzyme that cleaves AβPP to Aβ [69]. Another enzymatic target for Aβ production is γ-secretase, the catalytic component of presenilin-1 (PS-1) and glycogensynthase kinase-3β (GSK-3β), which decreased when human neuroblastoma SHSY5Y cells were treated with CUR, suggesting that CUR decreased Aβ production by inhibiting GSK 3β-dependent PS-1 activation [70].

In addition to inhibiting Aβ production, CUR also inhibits the aggregation of fibrillar Aβ in vivo and in vitro and promotes disaggregation. Reinke and Gestwicki [71] examined the presence of hydrophobicity, keto or enol rings, two phenyl groups and polar hydroxyl groups on the two aromatic rings of CUR for inhibition of amyloid aggregation. The two polar hydroxyl groups present at both extremes, capable of taking part in hydrogen bonding with polar pockets of the Aβ peptide are crucial for destabilizing β-sheets and disintegratingAβ dimers. 

The neuroprotective effects of CUR are not only limited in preventing the formation of Aβ fibril and its aggregations, but it also prohibits Aβ-mediated neurotoxicity. The in vitro study on human neuroblastoma SHSY5Y cells showed that CUR attenuates Aβ-membrane interactions, Aβ-induced membrane disruption, prevent intracellular calcium elevation and shift the Aβ aggregation pathway to the formation of nontoxic soluble oligomers and prefibrillar aggregates which stills requires in vivo study [72]. 

#### 3.1.2. Tau Inhibition

The risk of developing Alzheimer’s disease increases with age. Alzheimer’s disease usually begins with memory decline and later affects other cognitive abilities. Two different types of protein deposits are involved in the brain, namely “amyloid-beta plaques” and “Tau neurofibrillary tangles“ [73].

The appearance of your neurofibrillary tangles reflects the progression of the disease, they first manifest in the memory centers of the brain and then appear in other areas as the disease progresses. Tau proteins (or Tau aggregates) migrate along nerve fibers and thus contribute to the spread of the disease throughout the brain [74]. If proteins spread more quickly in the aging brain, this might explain why most people with Alzheimer’s disease are older [75].

The aggregation of hyperphosphorylated tau is crucial for AD pathogenesis and scientific studies have shown that CUR prevents tau hyperphosphorylation into NFTs [76]. The tau protein is phosphorylated after by phosphatase and tensin homolog (PTEN)/protein kinase B (Akt)/GSK-3β pathway induced by the GSK-3β enzyme, Aβ peptides which are inhibited by CUR to alleviate tau-induced neurotoxicity [77].

#### 3.1.3. Microglia Modulation and Neuro-Inflammation Inhibition

Decades of research have linked neuropathy with neuroinflammatory phenomena that can be provoked by microvascular damage, atherosclerosis, Aβ accumulation, age-related inflammatory factors and bacterial or viral infections that affect the BBB [78]. Any neurological damage/disorder can lead to microglial activation, followed by phenotypic proliferation and change [79]. In this process, Aβ diverts microglia from its neuroprotective phenotype to its neurotoxic phenotype. The neurotoxic phenotype express iNOS and major histocompatibility complex (MHC) II, activating the NF-κB pathway to produce several pro-inflammatory cytokines, such as TNF-α, IL-1β, IL-6, IL-12 and IL-23, and generate ROS and NO, which subsequently induce immune stimulation, neuroinflammation, the block of axonal remodeling and prevent neurogenesis. However, neuro- protective phenotypes mediate neuroprotection by Aβ phagocytosis and clearance, neuronal regeneration modulation, and arginase 1 (Arg1) release for tissue remodeling, wound healing and debris clearance [80]. 

Indeed, CUR has been proposed as a potent anti-inflammatory agent, able to reduce many neuroinflammatory mediators and modulate the activation of microglial. In an in vitro study in Aβ-activated microglia, CUR improved microglial viability and suppressed the activation and blocked extracellular signal-regulated kinase 2 (ERK1/2) and p38 kinase signaling, reducing TNF-α, IL-1β, and IL-6, mRNA and protein levels production [81]. In addition, and despite the CUR stimulatory activity on anti-inflammatory cytokines production (i.e., IL-4 and IL-10), namely in lipopolysaccharide (LPS)- activated microglia, it also has the ability to upregulates the expression of suppressors of cytokine signalling (SOCS-1), whereas reducing the phosphorylation of Janus Kinase 2 (JAK2) and Signal Transducer and Activator of Transcription-3 (STAT3). Thus, by preventing microglial inflammatory responses and inhibiting the plaque accumulation, the neuroprotective potential of CUR is enhanced, besides its direct effect on neuroinflammatory reactions by eliciting anti-inflammatory responses in microglia through JAK/STAT/SOCS signalling pathway modulation [82].

Liu et al. [83] demonstrated the ability of CUR to activate the peroxisome proliferator-activated receptor-γ (PPARγ) and amplify the PPARγ protein, which downregulates the NF-κB pathways. In addition, CUR reduced microglia and astrocytes activation, and cytokine production responsible for neuroinflammation. Besides this, when the isolated microglia of AD patients were treated with curcuminoids, Aβ phagocytosis by microglia raised by 50% compared to the control group suggesting activation of neuroprotective microglia phenotype [84]. Thus, preventing microglial inflammatory response, and inhibiting the plaque accumulation, CUR enhancement of neuroprotective. 

#### 3.1.4. Antioxidant Potential

In general, neuronal inflammation is a protective response for numerous cellular and tissue injury [85]. It represents a complex of local and general reactions of the body, which includes alternative phenomena, changes in vascular dynamics, proliferative events and finally, reparative phenomena [86]. But when the inflammation is uncontrolled, the effect initiates an excessive injury of cells and tissues, causing the destruction of healthy tissues and the occurrence of chronic inflammation. Inflammatory brain diseases, including Alzheimer’s disease and Parkinson’s disease, are characterized by an imbalance of redox status but also by chronic inflammation, the primary cause of injury, and cell death [87]. Reactive oxygen species (ROS) are recognized as key mediators of cell survival, proliferation, differentiation, but also apoptosis [88]. Excessive production of ROS (also known as oxidative stress) by mitochondria and NADPH oxidase is recognized as responsible for tissue injury associated with brain damage, inflammatory processes and neurodegenerative diseases, such as Alzheimer’s disease [89].

**Figure 4:**
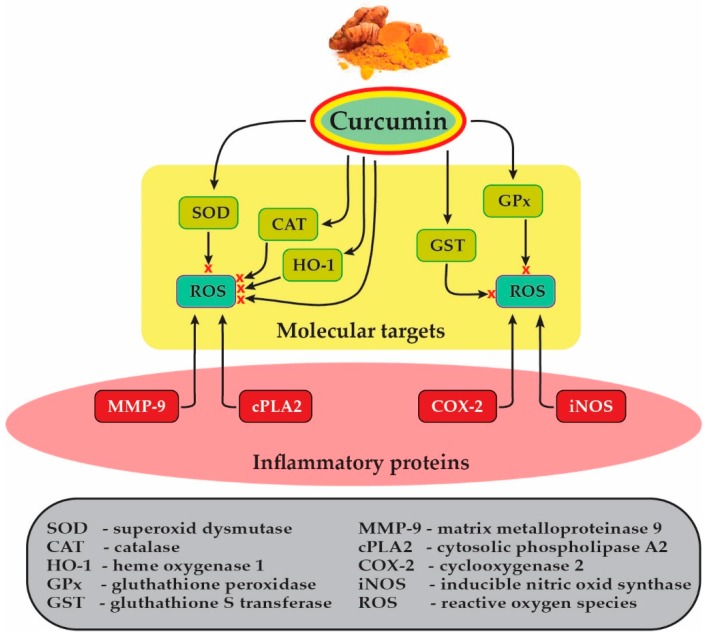
The molecular targets, anti-inflammatory and antioxidant mechanisms of Curcumin on the cells of the nervous tissue.

Many of the well-known inflammatory proteins, including matrix metalloproteinases-9 (MMP-9), cytosolic phospholipase A2 (cPLA2), cyclooxygenase 2 (COX-2), inducible nitric oxide synthase (iNOS), and adhesion molecules, are associated with oxidative stress (ROS generation) induced by proinflammatory factors (cytokines, peptides, infectious factors, peroxides). (Figure 4) Nerve cells, especially neurons, are susceptible to the adverse effects of oxidative stress. Numerous studies have concluded, the release of various inflammatory mediators by astrocytes and microglia, in response to oxidative stress [90].

Due to the demanding metabolic rate, increased oxygen demand, lower enzymatic defense against free radicals, and composed of easily oxidized lipids, the brain is particularly vulnerable to oxidative damages. The imbalance in the redox state with ROS accumulation or a decrease in antioxidant defense is linked to the development and progression of neurodegenerative diseases [91]. In AD, an excess of ROS may be produced by mitochondrial dysfunction, aggregation of Aβ proteins, phosphorylation and polymerization of tau and/or anomalous buildup of transition metals fostering the progression of the disease [92].

CUR has shown potent antioxidant activity either by scavenging the free radicals or upregulating cytoprotective mediators. Inhibited of lipid peroxidation or reduction of ferric ions, CUR has displayed comparable antioxidant activity with standard antioxidants [93]. Moreover, CUR can also scavenge superoxide anions (O_2_^−^) and hydroxyl radicals (OH^−^), and upregulate the expression of genes encoding for antioxidant proteins, such as catalase (CAT), heme oxygenase-1 (HO-1) and superoxide dismutase (SOD) [94,95]. In the brain, CUR can stabilize antioxidant enzymes, including SOD, glutathione peroxidase (GPx), glutathione S-transferase (GST) and protect for radical-induced DNA damages in neuronal cells [96,97].

#### 3.1.5. Neurogenesis and Synaptogenesis Promotion

The human brain is an organ capable of amazing activity in terms of its organization as a result of learning and experience [98]. This extraordinary property called neuroplasticity manifests itself in three main hypostases: during human development from the newborn stage to old age, during learning and during recovery after a neurological injury or disease at the sensory, motor or cognitive level [99].

There are several mechanisms of neuroplasticity, including Hebb’s law, synaptic plasticity, synaptogenesis, axonal growth and regeneration, growth factors, neurogenesis [100]. Synaptogenesis is a phenomenon by which neurons send new extensions that, by meeting the extensions of other neurons, form new synapses. Synaptogenesis begins *in utero* and continues after birth. It is carried out sequentially and has been followed especially at the level of neuromuscular junctions [101].

In the vicinity of a striated muscle fiber, the emergence cone (growth) of the axon begins to flatten and adhere to its surface, without any specialization being observed at the level of the two cell membranes. In an immediate next step, synaptic vesicles appear in the axonal end, and on the surface of the muscle fiber–acetylcholine receptors, distributed diffusely.

The accumulation of receptors in the area of the future postsynapse occurs under the influence of agrin, secreted by the neuron and fibroblast growth factor (FGF-β) from the extracellular environment; other molecules secreted by the neuron stimulate the activity of acetylcholine receptor-encoding genes in the juxtasynaptic nuclei [102].

The onset of synaptic activity, by generating action potential, inhibits gene activity for the same receptors in extrasynaptic nuclei. Later, there is a concentration of calcium channels in the presynaptic area, determining its intracellular growth, with a role in the organization of the cytoskeleton. Contact with the target is essential in synaptogenesis [103].

Multiple literature reports indicate the potential risk of synaptic damage and neuronal death due to declining neuronal growth factors and supporting factors, such as platelet-derived growth factor PDGF [104]. The in vivo study on animal models showed that the CUR-containing diet increases the level of neurotrophic factors and promotes neurogenesis, synaptogenesis, and improved memory functions [105]. Specifically addressing the effects of CUR on neural cells, it has revealed to have a great potential to limit histone acetylase activity and to promote neurogenesis, thus exerting a high impact on longevity and slowing down aging [106].

#### 3.1.6. Metal Chelation

In addition to tau and Aβ, an imbalance in metal homeostasis can induce misfolded protein aggregation and promote neurological diseases such as AD. Studies have suggested that metal ions stimulate the processing of AβPP, BACE1, and mRNA and promote the misfolding of Aβ oligomers [107]. Chemically, CUR is an excellent metal chelating ligand due to the presence of OH groups and one CH_2_ group [108]. CUR effectively chelate copper (Cu), iron (Fe), and zinc (Zn), making them unavailable to induce Aβ aggregation. Furthermore, CUR reduces expression of NF-κB levels induced by heavy metals in the neuroinflammation process [109].

## 4. Nanoformulation: Molecules, Extraction Techniques and Alzheimer’s, and Brain Diseases Effects

Initially, for CUR extraction from turmeric dried roots, a liquid-solid extraction procedure needs to be used [110]. By vacuum filtration or by gravity, the insoluble material is separated from the soluble one (CUR), which is extracted into the solvent. The solution obtained can be used in its liquid form, or instead, the solvent can be evaporated to recover the extracted material in crystalline powder [110].

In a study reported by Mandal et al. [111], a better method of extraction was discovered using the extraction process with ultrasound. The researchers found that ultrasound utilization in CUR extraction was much faster, just 70 min, compared to the liquid-liquid extraction process that required many hours. In addition, this method allows for obtaining a larger amount of CUR from the turmeric root. This study highlighted that this is a method who can be used in an effective way to reduce long botanical extraction times to a few minutes, non-thermic, without using heat [111].

Thus, the shorter and better extraction methods of CUR open new windows in the research of the phytotherapeutic actions of CUR such as: cholagogue action (stimulates bile release), anticancer, inhibition of the development of cancerous tumors and metastases (breast, stomach, colon, lung, liver, skin) [112,113,114,115], anti-inflammatory action, hepatoprotective inclusive in non-alcoholic fatty liver disease [116], antiatherosclerotic, antiplatelet effects but not in cases of vitamin K coagulopathies [117], prevents AD and other neurodegenerative disorders [118,119].

However optimal therapeutic results cannot be achieved due to its poor solubility, low gastrointestinal absorption with a reduced bioavailability [120]. An important objective for the researchers was to increase the bioavailability of CUR, especially its polyphenols with a low absorption rate and an increased liver metabolism rate. This low absorption is correlated with the size of the polyphenols, as they cannot penetrate through the intestinal barrier, and with their low solubility in both water and lipids [121].

It was observed an increased affinity of CUR polyphenols for phospholipids that are both hydrophobic and hydrophilic and act as emulsifying agents, increasing bioavailability and consequently, the researchers decided to associate the two types of substances [122]. Because the most abundant phospholipid in the human tissues is phosphatidylcholine, it was chosen to use it, with the role of improving both the absorption of polyphenols in the intestine and their penetration into the cell [123,124], so the CUR phytosomes were obtained, as a special CUR formulation with a curcuminoid bioavailability of up to 29 times higher compared to the simple form of CUR [124].

The challenges of increasing CUR bioavailability have continued and culminated with the development of nanoparticles. In recent years, with the development of nanotechnologies [125], nanoformulations have made it possible to develop nano curcumins (CUR encapsulated nanoparticles). Different types of CUR nanocarriers such as liposomes, solid-lipid nanoparticles, micelles, polymer nanoparticles, and polymeric conjugates have been developed to the treatment of different disorders, among them neurodegenerative disorders.

Nanocurcumin is the result of compression of the bulky CUR molecule at less than 100 nm with higher bioavailability properties. A lot of new technological methods have been developed to design nanoparticles with CUR who have an increased bioavailability [126]. The CUR nanoparticles are effective ways of administering of some drugs due to their increased bioavailability and superior cellular absorption characteristics. Nanocurcumin can also be obtained only from filtered CUR without using the nanocarrier conjugates. CURdissolution can be achieved with ethanol and then homogenized under high pressure with water containing 0.1% citric acid [127].

Cyclodextrin-CUR inclusion complexes are composed of cyclic oligosaccharides formed from a variable number of glycopolymeric monomer units, which varies between six to eight units. These contain a lipophilic central cavity and a hydrophilic external layer [128]. Of the three different types of cyclodextrins (α, β, γ), β-cyclodextrins have been widely used, since they are easily accessible and cost-effective, improve stability, reduce bitterness, improve water solubility and bioavailability. Using solvent evaporation or pH change technologies, CUR is conjugated with β-cyclodextrins in order to obtain inclusion complexes for increasing the CUR absorption [128].

Microspheres andmicrocapsules with encapsulated CUR or dispersed in polymeric particles (camptothecin, routine, zedoaric oil) form microscopic spheres/capsules that significantly increase bioavailability and pharmacological efficacyin the target organs, especially the brain [129]. The microcapsules were formulated with a layer and the CUR was incorporated into hollow microcapsules with polyetheresis of the ectrolytic multilayer. Studies highlighted that microcapsules andmicrospheres had demonstrated a remarkable increase in CUR’s stability and bioactivity [129].

Liposomal CUR (liposomes) are closed round particles, phospholipids, with CUR included into an aqueous interior, widely used as nanocarriers to increase CUR absorption and efficacy [130,131]. Strong migraine headaches experimentally induced in mice were successfully treated by the combination of sumatriptan with intravenous liposomal CUR at doses of 2 mg per 100 g body weight [47]. Recently, several changes in polymeric conjugate liposome CUR have developed to achieve better clinical outcomes in AD [132].

The CUR polymeric micelles represent another nanocarrier of CUR that significantly increases its low solubility, poorbioavailability and stability characteristics [133]. In a recent study, CUR was encapsulated in cationic micelles such as dodecyl trimethyl ammonium bromide or cetyltrimethyl- ammonium bromide with increased CUR loading capacity, increased solubility, reduced toxicity and decreased metabolic degradation [133].

CUR microemulsions are small drop dispersions (1–100 μM size) of isotropic oil and water mixtures stabilized using the interfacial films of the surfactant molecules [134]. These microemulsion systems are the pharmaceutical forms used for the administration of hydrophobic drugs. Advantages of lipid CUR microemulsion include improved CUR dissolution, thermodynamic stability and superior solubility. Tween-20 as emulsifier and triacylglycerol were used to formulate the microemulsion droplets under rapid and high-pressure homogenization procedures [134].

Solid dispersion with CUR involves dispersing it in a non-pharmacological solid nanocarrier or matrix using the solvent melting technological process [135]. Recently more nanoformulations have been designed to obtain crystalline and amorphous solid dispersions that have been shown to remarkably enhance CUR physicochemical and pharmacokinetic activities [135,136]. These include wet melting and freeze-drying.

The CUR nanogels made up of three-dimensional hydrophilic polymer networks that can absorb large amounts of water or physiological fluids internally while maintaining the internal structure of the network [137]. These nanogels are an effective CUR release formulation, high dispersibility stability, CUR release efficiency, and rapid release.without histopathological changes of nasal mucosae. CUR has the great advantage of being easily encapsulated inside of a nanogel [137].

CUR solid lipid nanoparticles (SLNs) are composed of natural lipids such as lecithins or triglycerides that remain solid at normal temperature (37 °C). Theseprotects labile compounds from chemical degradation and can improve the bioavailability of CUR with increased cellular absorption [138].

CUR polymer nanoparticles have nanometric dimensions, are highly biocompatible and circulate slightly in the blood for a more extended period of time. Some of the widely used synthetic polymeric conjugates include chitosan (CS); poly(lactic-co-glycolic acid) (PLGA) [139], polyethyleneglycol (PEG) [140] and hydrophobically modified starch. PLGA with PEG-5000 carrier stabilizer was used to design CUR nanoparticles with an efficiency of 97.5%, 81 nm diameter. Experimental studies have shown that PLGA loaded CUR nanoparticles have higher cellular absorption andincreased bioavailability [139] and easily crossing the hematoencephalic barrier with brain release of CUR with a beneficial effect in neurological diseases [141].

More recently, CUR-encapsulated exosomes have been the target of increasing attention, as they have shown to be of great interest, namely on neural therapy, besides to have a high bioavailability and safety, have ability to stimulate the immune system, and able to reach high concentrations in blood [142]. Recent experimental studies on rats have highlighted the efficacy of curcumin treatment encapsulated in exosomes, vesicles with tiny membranes in Alzheimer’s disease [142]. Curcumin encapsulated in exosomes crosses the BBB and reaches the neuronal tissue where it inhibits the hyperphosphorylation of Tau proteins, thus reducing the symptoms of Alzheimer’s disease. This mechanism is explained by the activation of the AKT/GSK-3β pathway [143].

Magnetic CUR nanoparticles can be used in neurodegenerative disorders under the influence of external magnetic fields [144]. CURinclusion into Fe_3_O_4_-CUR conjugate with oleic acid or CS on the outside results in the formation of water-dispersible fluorescence magnetic nanoparticles with increasing cellular absorption andincreased bioavailability [144]. All these nanoformulations which have the main purpose increasing of CUR’s bioavailability have beenused as alternative therapies in neurodegenerative diseases such as AD [145], PD and MS [30].

### 4.1. Alzheimer’s disease

**Figure 5:**
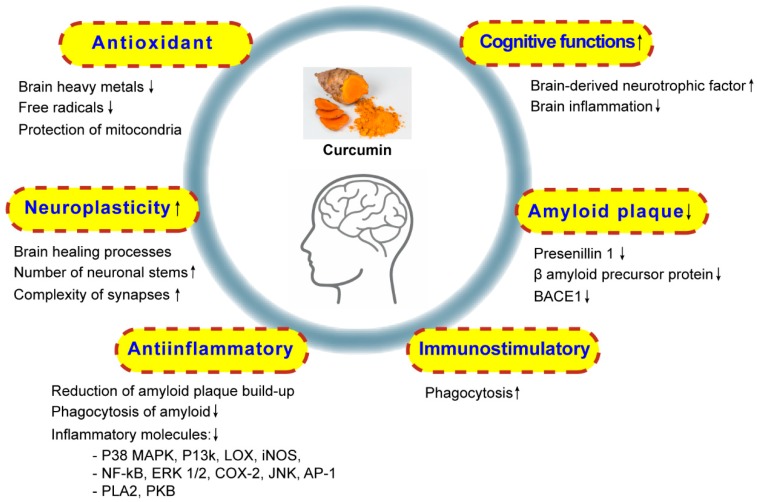
The mechanism of action of curcumin nanoformulations in Alzheimer’s disease [142,143,146,147]. Legend: p38 mitogen-activated protein kinase (p38 MAPK), phosphatidylinositide 3-kinase (PI3K), 5-lipoxygenase (5-LOX), inducible nitric oxide synthase (iNOS), Tumor necrosis factor-alpha (TNF-*α*), nuclear factor-*κ* B (NF-kB), extracellular signal-regulated kinase 1/2 (ERK1/2), cyclooxygenase 2 (COX-2), c-Jun N-terminal kinase (JNK), activator protein-1 (AP-1), phospholipase A_2_ (PLA_2_), protein kinase B (PKB, also named Akt), β site amyloid precursor protein cleaving enzyme 1 (β-secretase 1, BACE1).

Unfortunately, no really effective treatment is still available for AD. Therefore, prevention is first of all the most important. CUR, formulated as nanoparticles, can cross the BBB and act on brain cells, being active against various neurological diseases [146,147].The mechanisms of action of CUR in AD (Figure 5) have been proven by many studies [62].

#### 4.1.1. The Prevention of Amyloid Plaque Accumulation

The active substance in turmeric, CUR, blocks β-amyloid plaques formation throughdifferentways [84,148]. On the one hand, CUR blocks PS-1 which contributes to plaque formation and, on the other hand, it reduces the accumulation of amyloid formations [70]. Due to their ability to reduce BACE1, there is the possibility of developing a CUR treatment for AD [149].

Bukhari et al. [150] have shown that CUR derivatives inhibit protein accumulation in the dementia-affected brain, thus reducing their neurotoxicity. A recent study demonstrated a significant decrease in amyloid plaque because free CURcan cross the BBB and attaches to neurotoxic proteins, preventing them from sticking together and forming amyloid plaques between neurons [151].

#### 4.1.2. Antioxidant Mechanism of Curcumin in Alzheimer’s

With physiologic aging, the brain accumulates metal ions (Fe, Zn and Cu) and inflammation and accumulation of amyloid plaques are triggered. It has been found that CUR derivatives inhibit the accumulation of heavy metals in AD [152]. A study conducted by Fan et al. [153] analyzed the role of different antioxidants in foods, including CUR, in AD. The result showed that due to its chelating action, CUR prevents the accumulation of heavy metals in the brain.

#### 4.1.3. Neuroplasticity Stimulation by Curcumin Nanoparticles

The brain has a fantastic recovery capacity, called neuroplasticity [154]. CUR nanoparticles have shown that this therapy induces a regeneration process of neurons. Fan et al. demonstrated in an experimental study on mice that CUR reduces the dysfunction of neuronal plasticity structures inducedviaIL-1β/NF-κBpathway [155]. A study by Hucklenbroich et al. [156] revealed that a turmeric compound, aromatic-turmerone, can be a promising aid in regenerating neurons. It was also shown that when neuronsare exposed to aromatic-turmerone, neural stem cells grow in both number and complexity, characteristic of the healing process. This effect was seen also in vivo on a rat model that, after exposure to aromatic-turmerone, increased their stem cell production and generated healthy new brain cells [156].

#### 4.1.4. Reducing Neuroinflammation

In AD, neuroinflammation is associated with exposure to toxic agents, infections, or amyloid plaque formation [157]. An experimental study performed by Yang et al. [158] on transgenic mice showed that CUR may bind to amyloid plaques when injected through the carotid artery.CUR caninhibit many molecules (Figure 4) involved in inflammation [159] and can cross the BBBthus reducing neuroinflammation [158]. CUR reduces inflammation from AD and prevents the formation of amyloid plaques. It also acts on the kappa-B factor, a macro protein involved in the regulation of inflammation [160] and translocation produced by IL-1β and subsequent expression of NF-κB determined by pro-inflammatory genes [161].

#### 4.1.5. Curcumin Supports Cognitive Function and Memory

CUR protects nerve cells and increases memory skills and learning [105]. The mechanism is provided by a study of Xu et al. [162] on a rat model, according to which CUR increases the level of BDNF, a protein that supports the activity of neurons. A placebo-controlled trial conducted by Rainey-Smith et al. [163] analyzed the effect of a CUR supplement on a group of elderly people over a period of one year. Cognitive decline was found in different stages in the placebo group, but not among those who received CUR [163]. Another study conducted by Small et al. [164] showed that CUR can improve mood and memory in the case of patients with mild loss of memory related to age.

CUR is easily absorbed in people who have memory problems without dementia, emphasizing good results on microscopic plaques and nodules in the brain in patients diagnosed with AD [164].

It is not known exactly how the effects of CUR are produced, but it may be due to its possibility to decrease brain inflammation that has been correlated to AD and major depression. These actions were demonstrated in a double-blind, placebo-controlled study that included 40 volunteer patients aged 50–90 years with mild memory problems. Subjects were randomly assigned to the placebo or group treated with 90 milligrams of CUR twice a day for 18 months. 40 subjects were evaluated using standardized cognitive tests at the start of the study, and after 6-month intervals. Blood CUR concentration was controlled at the start of the study and after 18 months [164]. The results show that those who took CUR have significantly improved their memory and attention, while subjects receiving placebo did not show any improvement. In memory tests, people who took CUR improved 28% in the 18 months and increased their mood.On the other hand, their brain scans showed fewer signs of amyloid and your tonsillitis and hypothalamus than those who took a placebo. Only four people who took CUR and two of those taking placebo experienced mild side effects (abdominal pain and nausea) [164].

#### 4.1.6. Immunostimulatory Effect

AD is also characterized by dysfunction of the immune system [165]. In the normal brain, the innate immune system is supported by astrocytes and microglial cells that clean the amyloid plaques, but this is not the case also for AD [166]. A study led by Teter et al. [167] demonstrated in mice that natural CUR derivatives restore immune function and stimulate immune cells to eliminate amyloid plaques by phagocytosis (a process by which phagocytes swallow damaged cells and other residues). In another experimental study, researchers isolated immune cells from Alzheimer’s patients and treated them with curcuminoids. The ability of these cells to remove amyloid particles has increased due to CUR [168]. The results suggest that curcuminoid therapy could be applied as immunotherapy in AD.

### 4.2. Parkinson’s Disease

The difficulty of movement is one of the main PD-associated symptoms. α-Synuclein protein leads to rigidity in locomotion [169]. It is demonstrated that the simple administration of CUR can bring a significant improvement in walking in PD patients. A recent study demonstrated that CUR manages to attenuate PD-related deficits by increasing the level of antioxidant enzymes. By reducing oxidative stress, CUR slows the loss of motor function and increases the life span [169]. Kundu et al. [170] have demonstrated on a rat model of PD that nanocarriers with CUR and piperine in the form of nanoparticles formulated with glyceryl monooleate improve their penetration into the brain by crossing the BBB. Increasing the bioavailability of CUR released from nanoparticles has reduced the synuclein fibrils by reducing their aggregation, thus decreasing dopaminergic neuronal degeneration and improving motor coordination [170].

### 4.3. Multiple Sclerosis (MS)

MS is a chronic autoimmune disease that affects the brain, spinal cord, and optic nerves and it is caused by demyelination of myelin plaques and inflammatory infiltrations in brain [171]. The main symptoms observed are loss of vision or double vision, loss of balance, stiffness, tiredness, difficulty in speaking and/or swallowing, intellectual deficits, changes in emotional states [171]. Unfortunately, there is no complete treatment for MS but CUR can be a good alternative therapy to relieve these symptoms, according to in vivo study conducted by Natarajan and Bright [172]. These researchers used an animal model of MS, represented by experimental allergic encephalomyelitis (EAE) autoimmune produced by injection of myelin in mice. To these EAE mice were administered parenterally 50–100 micrograms of CUR three times a week for one month. CUR-treated mice showed very few MS symptoms; and mice that did not receive CUR were paralyzed by the disease within 15 days. At the study end, CUR-treated mice (100 µg) exhibited imperceptible symptoms of the disease [172]. This effect is explained by the inhibition of IL-12, one of the main causes of myelin plaque damage [172].

In a recent experimental study on Lewis rats with induced EAE it was demonstrated that polymerized nanoparticles with CUR administered in doses of 12.5 mg/kg favor the remyelination process of neurons by repairing of myelin sheaths and reduces neuroinflammation by inhibiting the following pro-inflammatory genes: NF-ββ, IL-17, IL-1, TNF-α, and monocyte chemoattractant protein-1 (MCP-1) [173].

## 5. Nanocarrier Formulations for Curcumin Delivery

Despite CUR’s therapeutic efficacy in neurological and brain diseases, a number of drawbacks still remain for its briader use, such as its poor bioavailability and low cellular uptake (low tissue levels) [174]. Those critical issues, that reduce its clinical application, are mainly connected to the low absorption and rapid systemic elimination (hepatic metabolism) [175]. Moreover, the high hydrophobicity and low solubility of CUR in the water at acidic (or neutral) pH cause a major limitation for their wider therapeutic use [175]. It is well known that nanocarrier-based delivery systems can enhance the drug colloidal stability as well as its ability to cross the biological barriers and to reach the targeted regions [176,177]. For this reason, the employment of the nanocarriers as delivery systems for CUR represents a promising approach for the development of nano-platforms dedicated to the treatment of neurological disorders and brain diseases [177,178].

**Figure 6:**
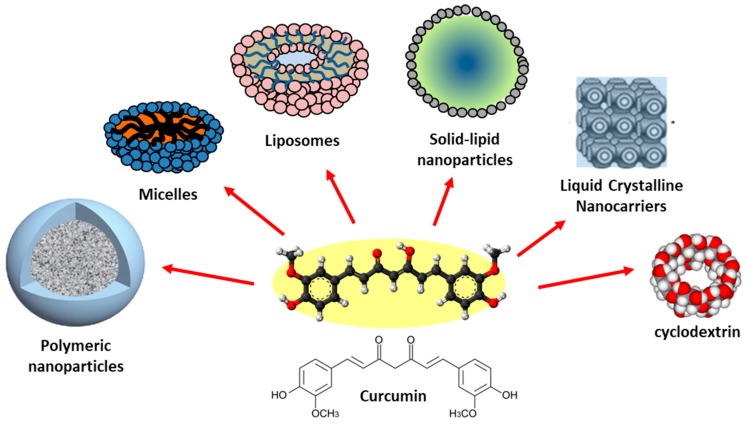
Main types of CUR-based nanocarrier formulations for the treatment of Alzheimer’s and brain diseases.

However, the treatment of the human neurological and brain diseases requests that the delivery of CUR happens at specific target sites, by overcoming the complex microenvironment of the BBB. Within these general issues, recent progress in drug delivery approaches stimulated the development of novel nanocarriers encapsulating CUR, such as polymer nanoparticles (and micelles), lipid-based (and liposome) nanocarriers, liquid crystalline nanocarriers (LCNs) andcyclodextrins (Figure 6).

CUR encapsulation into nanocarriers improves not only its bioavailability and solubility, but also increases its colloidal stability by protecting it from the influence of the tissue micro-environment, thus enhancing the sustained release of CUR at target sites. There are a number of key factors that govern the distribution parameters of drugs within brain tissues, including the plasma protein binding, drug–tissue interactions (and binding) in the brain region, blood (influx/efflux) flow rate at the BBB level and the rate of drug metabolism in the brain region.

Nowadays, newer formulations of nanocarriers are designed with the aim to achieve efficient transport and improved the therapeutic efficacy of CUR. In this section, we describe the main types of formulated CUR-based nanocarriers and their effectiveness in specific drug delivery processes.

### 5.1. Polymer-based Nanocarriers: Polymer Nanoparticles and Micelles

Nanocarriers composed of biocompatible and biodegradable polymers nanoparticles are of high interest as drug delivery systems, given their versatility in boththerapeutic drug preparation and delivery into target tissues. Polymers employed have distinctchemical-physical characteristics, and the modification of their chemical groups has been used for functionalization and drug conjugation of many polymer-based nanocarrier platforms, increasingly employed for therapeutic drug delivery processes at the central nervous system (CNS) level [178].

Among the variety of polymer-based systems, PLGA is the most commonly-used biocompatible polymer for the treatment of neurological disorders [179]. A recent investigation analyzed different CUR-loaded PLGA nanoformulations developed with the scope to improve the kinetics of tissue distribution and BBB penetration, in a freely-moving rat model [180]. In that case, CUR exposure in the body was increased with either intravenous or oral nanoparticle administration and the relative bioavailability of CUR-loaded PLGA formulations showed a 22-fold increase over conventional CUR [180]. In another study, a sensitively prolonged retention time of CUR in the cerebral cortex (increased by 96%) and hippocampus (increased by 83%) was observed with the PLGA nanoparticle encapsulation [181]. Furthermore, neuronal uptake and neuroprotective effect of CUR-loaded PLGA nanoparticles have been recently investigated, in vitro, to the human neuroblastoma SK-N-SH cells [182]. In that case, the CUR-loaded PLGA nanoparticles were able to protect human neuroblastoma SK-N-SH cells against H_2_O_2_-induced oxidative damage thus evidencing a promising (nontoxic) drug delivery method to protect neurons against oxidative damage as observed in AD [182]. Moreover, CUR-encapsulated PLGA 50:50 nanoparticles (NPs-Cur 50:50) displayed higher antioxidant and anti-inflammatory activities than free CUR, and have evidenced the ability to prevent the phosphorylation of Akt and tau proteins in SK-N-SH cells induced by H_2_O_2_ [183].

Poly(butyl)cyanoacrylate (PBCA) nanoparticles represent another interesting polymer-based nanocarrier for CUR drug delivery [184,185]. Preparation of apolipoprotein-E3 (ApoE3) mediated PBCA nanoparticles (ApoE3-C-PBCA) containing CUR evidenced an enhanced (in vitro) apoptosis- induced anticancer activity against SH-SY5Y neuroblastoma cells [184], compared to CUR plain solution and CUR loaded PBCA nanoparticles (C-PBCA), with apoptosis being the underlying mechanism [184]. Moreover, in vitro cell culture study revealed an enhanced therapeutic efficacy of ApoE3-C-PBCA nanoparticles against β-amyloid induced cytotoxicity in SH-SY5Y neuroblastoma cells compared to CUR solution [185].

Finally, CS, a natural linear biocompatible and biodegradable polysaccharide, represents another interesting polymer for brain delivery purpose, due to its low toxicity and immunogenicity. CS polymer contains primary amine groups, which make CS-nanocarriers positively charged vehicles and efficient tools for brain therapeutic interventions [186,187,188]. CUR inclusion into CS nanoparticles improves its chemical stability, prevents its degradation, and facilitates the uptake of the cell membrane and the (controlled) release of CUR [186]. In a recent investigation, CUR-conjugated CS nanoparticles with diameter < 50 nm evidenced efficacy against arsenic-induced toxicity in rats [187], while CUR-loaded CS–bovine serum albumin nanoparticles showed their efficacy in the enhancement of Aβ 42 phagocytosis and modulated macrophage polarization in AD [188].

Efficient release of the encapsulated CUR and improved bioavailability can also be achieved by means of the polymer micellesnanocarriers, obtained by the self-assembly of amphiphilic polymers. In this case, the micelles hydrophobic core creates a microenvironment for the incorporation of the hydrophobic CUR, while the hydrophilic shell ensures their water solubility. Particularly interesting is the use of PEG for the hydrophilic polymer block, as it creates a local surface concentration of highly hydrated polymer brushes [189,190,191] that sterically inhibit the interactions with plasma proteins (or cells), thus reducing the nanocarrier uptake process by mononuclear phagocytic systems (MPS). Moreover, the surface coatings with PEG blocks have been shown to increase colloidal stability, solubility as well as to improve polymeric nanoparticle diffusion in brain tissues [191,192]. Recently, the in vivo investigation of CUR-loaded PLGA-PEG micellar nanocarriers evidenced an increase of the relevant pharmacokinetic parameters [193]. More specifically, the CUR-loaded nanocarriers could overcome the impaired BBB, efficiently diffuse through the brain parenchyma, and deliver a protective effect in the regions of injured neonatal rats’brains (with hypoxic-ischemic encephalopathy) [193]. Moreover, micelles formed by synthetic PLGA-PEG-PLGA triblock copolymers have been revealed to be able to modifyCUR pharmacokinetics and tissue distribution [194].

It is worth pointing out that the use of simple PLGA nanocarriers without further modifications presents a number of intrinsic drawbacks, connected with the short blood circulation time and the difficulty of passing through the BBB. After crossing the BBB, the polymer nanocarriers must then overcome and penetrate the brain tissues and diffuse some distance before diseased cells can be targeted. For this reason, cell-specific delivery can be promoted by the surface modifications on the polymer nanocarriers with targeting ligands or peptides. Recently a variety of approaches have been developed for the engineering of modified PLGA nanocarriers (with targeting ligands) for enhanced drug delivery to the brain and the CNS [179]. For example, in vitro investigation of CUR loaded-PLGA nanoparticles were conjugated with Tet-1 peptide for potential use in vitro in AD [195], and with glutathione that is able to modify the route of internalization (enabling them to escape the uptake through micropinocytosis) toward a safer pathway and avoiding the lysosomal degradation [196]. Furthermore, an innovative PLGA nanocarrier was designed and the results evidenced that compared to other PLGA nanoparticles, CRT peptide modified-PLGA nanoparticles (co-delivering S1 and CUR) exhibited enhanced beneficial effect in AD treatment in mice [197].

Finally, it is worth pointing that mixed polymeric micelles provide an interesting alternative approach for the formation of CUR delivery systems, due to enhanced colloidal (long-term) stability, drug-loading capacities compared with simple polymer micelles [198]. Moreover, mixed micelles can provide addition (and multiple) functionalities by the constituent copolymers to the micellar nanocarrier, thus increasing their performances in the involved drug delivery process [199]. In conclusion, polymer micelles encapsulation of CUR provides a sensitive increase in solubility and bioavailability, making this formulation very promising for the development of therapeutic tools for AD and brain disease clinical applications.

### 5.2. Lipid-Based Nanocarriers

Lipid-based nanocarriers represent a versatile nanomaterial platform to develop enhanced drug delivery systems formultiple applications in nanomedicine and biotechnology [200,201]. Synthetic or natural lipids are more biocompatible than the polymeric and inorganic nanocarriers, and they present a marked ability to penetrate the BBB even without any specific functionalization (passive targeting). Moreover, mixed lipid-based systems present enhanced colloidal stability as well as a wide range of morphological and structural properties generated by a versatile self-assembly process, as evidenced by various structural investigations [201,202]. The formation of lipid-based nanocarriers is controlled by specific soft interactions that regulate the colloidal stability of therapeutic drugs in a harsh bio-environment of diseased tissues, thus allowing better control over drug release kinetics [203]. SLNs [204], nanostructured lipid carriers (NLCs) [205] and liposomes [200], are the most important representatives of the lipid-based nanocarriers, that have been used for the treatment of brain diseases in the last decades.

#### 5.2.1. Solid Lipid Nanoparticles and Nanostructured Lipid Carriers

Due to their inherent ability to cross theBBB, SLNs and NLCs are drug delivery systems that have been used for the active and passive targeting treatment of a variety of brain cancers and neurodegenerative diseases [206,207]. SLNs present a small spherical shape (with a radius ranging between 50 to 200 nm) with a (lipid matrix) solid core at the body (and room) temperature. They are composed of a mixture between different lipids and amphiphilic molecules (about 1–5% w/v of surfactants/cosurfactant) that stabilize the lipid core region. The glyceride derivatives (such as the monoglycerides, triglycerides, and complex glyceride mixtures), which are easily assimilated by our metabolism, are generally the most abundant component. SLNs represent an efficient drug delivery system for both lipophilic and hydrophilic therapeutic drugs. Their specific composition, made of lipids and surfactants, strongly influence their physicochemical properties such as size (and polydispersity), colloidal stability, loading and release properties of the active drugs [204]. SLNs are able to naturally cross the BBB due to their highly lipophilic nature (passive targeting). Brain uptake is performed by the paracellular pathway through the opening of the tight junctions in the brain microvasculature. Brain targeting with ApoE receptors, which is predominantly expressed in the brain, facilitated transport across the BBB (active targeting) [207].

With the aim to achieve an efficient and optimized CUR loaded nanoparticles with high drug payload, a preparation process of (small size) CUR loaded SLNs and NLCs was performed using experimental design and a multi-objective optimization approach [208]. The investigation evidenced that by the modulation of the key and control factors (such as the drug-to-lipid ratio, surfactant concentration, and homogenization rate), it is possible an experimental optimization of their effects on the nanoparticle size (and polydispersity) and loading efficiency. More specifically, the entrapment efficiency of CUR was found to be 82% (in SLNs) and 94% (in NLCs). The pharmacokinetic studies (after intravenous administration of 4 mg/kg dose of CUR in rat) evidenced that the amount of CUR available in the brain in CUR-loaded NLCs (AUC0-t = 505.76 ng/g·h), was significantly higher than the CUR-loaded SLNs (AUC0-t = 116.31 ng/g·h) and the free CUR (AUC0-t = 0.00 ng/g·h) [176]. Furthermore, CUR-loaded SLNs were investigated to assess their efficacy in the treatment of the BV-2 microglial cells against LPS-induced neuroinflammation [208]. The SLNs showed higher inhibition of NO production compared to conventional CUR in a dose-dependent manner. Moreover, the mRNA and proinflammatory cytokine levels were reduced in a dose-dependent manner in comparison to those with free CUR [208]. Finally, CUR-loaded SLNs exhibited a greater permeability than dietary CUR in vitro and showed marked effectiveness for AD therapy [209]. In that case, CUR has been shown to prevent Aβ 42-induced neuronal death by inhibiting ROS production or by blocking apoptotic death pathways and boosting cell survival pathways [209].

NLCs represent a slightly modified version of SLNs, where the structure of the solid lipid core contains imperfections in the crystal structure. This imperfect crystal, (obtained by mixing liquid/solid lipids and the addition of mono-, di- and triglycerides lipids with different chain lengths) increases the internal free space of the solid, thus resulting in a higher drug loading efficiencies [204,206]. A recent study showed that CUR-loaded NLCs significantly increase the accumulation rate of CUR in rat brain, as also its serum levels [210]. Their effects were evidenced by reduced oxidative stress parameters in hippocampal tissue and improved spatial memory. Moreover, histopathological studies revealed the CUR-loaded NLCs potential in decreasing the Aβ hallmarks in the animal model with AD. The neuroprotective potential of Cur-NLC in both pre-treatment and treatment modes also showed that loading CUR in NLCs is an effective strategy to increase CUR delivery to the brain and to reduce the Aβ-induced neurological abnormalities (and memory defects) [210]. A recent study evidenced that NLCs enhance the bioavailability and brain cancer inhibitory efficacy of CUR both in vitro and in vivo [211]. Observation of the time-dependent cellular uptake and ROS production, evidenced that CUR loaded NLC formulation not only improved the apoptotic induction effect of CUR, but markedly increased bio-availability and brain (and tumor) targeting effect, thus allowing trigger effect on the carcinoma digression [211]. Finally, both SLNs and NLCs can be produced byusing diverse formation methods, easily scaled up and without requiring the use of organic solvents, thus avoiding toxicity effects of the final product. The high-pressure homogenization technique (HPH) represents the most common technique due to its relatively low cost, and its ability for large-scale production [212].

#### 5.2.2. Liposomes

Liposomes are a highly versatile and biocompatible drug delivery system, with the potential for carrying different types of bioactive drugs and molecules across the BBB [213,214]. They consist of uni- or multi-lamellar lipid bilayers structures composed of phospholipids, with an internal aqueous core. They exhibit various architectures that depend on the preparation methods and the involved self-assembly process. Despite their intrinsic colloidal stability, depending on the conditions of the solution and the incorporated components, liposomes may aggregate and/or fuse together, thus changing their size or surface charge expression [215,216]. For this reason, liposome surface functionalization represents a widely used strategy to avoid the evolution of an inefficient drug delivery system and the degradation of the performances to the point of action. Liposomes can incorporate both hydrophilic drugs (entrapped in the aqueous core) and lipophilic (or hydrophobic) therapeutic compounds inserted in the hydrophobic region of the lipid bilayer.

Concerning the mechanism of action with biomembranes, the positively charged nanocarriers (cationic liposomes) can facilitate cell internalization and (nonspecific) uptake by electrostatic interactions with the negative charge of the endothelial cell membrane. It is worth pointing that charge expression of (bio-)membranes may induce complex aggregation behavior and morphological transition in the presence of soft nanoparticles or drugs macromolecules within the charged complex microenvironment of the biological systems [217,218]. In any case, once in the bloodstream, most liposomes are covered around their surface by a (plasma) proteins corona (e.g., fibrinogen, immunoglobulins), leading to MPS activation and successive liposomes removal from the bloodstream [213]. This circumstance causes a reduction of the sufficient number of liposomes that can be delivered to the brain tissues and consequently requests a higher dose to reach a sensitive therapeutic efficacy. In these cases, the surface-modified (targeted) liposomes are required for effective delivery across the BBB and to stimulate a (specific) molecular interaction for an effective therapy for AD [214,218,219]. The main efficient strategy for crossing the BBB is obtained by the functionalization of the liposome surface by using biomolecular ligands that enhance the BBB transport and targeting processes for AD therapy. It has been shown that liposomes modified with transferrin [220,221] and lactoferrin [222], the most commonly employed targeted receptors, could cross and penetrate the BBB via receptor-mediated endocytosis. Moreover, the surface functionalization of liposomes with PEG (stealth liposomes) cause a drastic reduction in the formation of the so-called proteins corona thus providing a longer circulation time and improvement of their pharmacokinetic profile [192,213]. These attractive properties of liposomes stimulate a growing interest in the development of suitable nanocarrier systems for the delivery of active drugs, such as CUR, that act on the CNS. Encapsulation of CUR with liposomes areused for therapies targeting Aβ in AD, through a contrasting action against the accumulation (and deposition) of plaques of Aβ peptide in the brain, with minimum side-effects, and the enhancement of CUR solubility and its cellular uptake [218,219].

Recently, liposome nanocarriers were bifunctionalized with both a peptide derived from the ApoE receptor-binding domain (for BBB targeting) and with phosphatidic acid (for Aβ-binding). The electron microscopy experiments evidenced that the bifunctionalized liposomes are able to disaggregate and hinder the formation of Aβ assemblies in vitro.These results evidenced the versatility of bi-functional liposomes in their tasks to destabilize brain Aβ aggregate, favor peptide removal across the BBB, and its final peripheral clearance [218]. Moreover, CUR-conjugated nanoliposomes with high affinity for Aβ deposits, recently found application in diagnosis and targeted drug delivery in AD. More specifically the nanoliposomes strongly labeled Aβ deposits in the post-mortem brain tissue of AD patients and in the APPxPS1 mice. The injection of the CUR-conjugated nanoliposomes in the neocortex and hippocampus of mice evidenced the ability to specifically stain the Aβ deposits in vivo [219].

**Figure 7:**
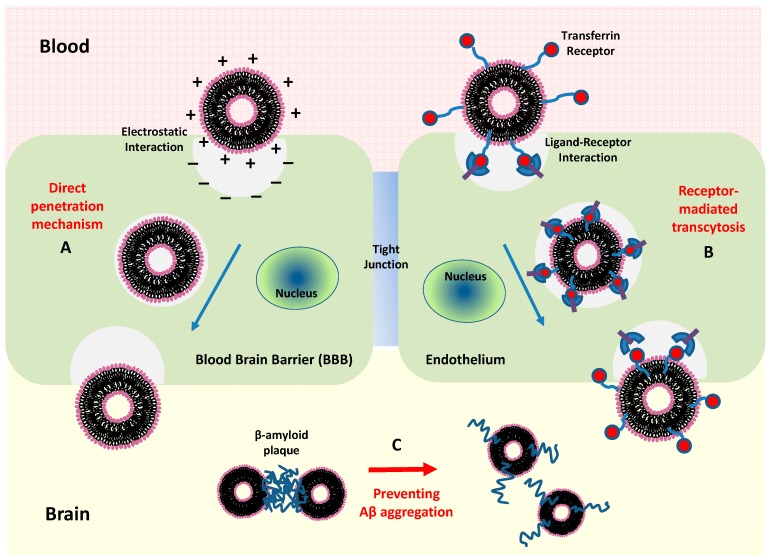
Transport process of liposome nanocarriers across the blood-brain barrier (BBB).In the direct penetration mechanism (**A**), liposome endocytosis is favored by the ionic interaction of positively charged liposome surface groups (due to the presence of cationic lipids, or positively charged (+++) amino acids) with the negative charge (− − −) of the endothelial cell membrane of the BBB. As an example, liposomes internalization may be favored by the negative charge exhibited by cell-penetrating peptides (e.g., CPPs TAT - transactivator of transcription of human immunodeficiency virus). Receptor-mediated transcytosis mechanism (**B**) exploits the specific interaction with receptors highly expressed at the BBB (e.g., transferrin receptor). Receptor-ligand binding interaction regulates both liposome internalization (crossing the BBB) and the delivery process of the liposome nanocarriers within the brain. Once that the liposome reaches the inside brain region, multi-functional liposomes can direct their action at the Aβ target, for AD therapy (**C**).

An alternative way to reach the brain tissue region consists of the exploitation of the (non-specific) interaction of liposomes with the BBB, by means of the cell-penetrating peptides (CPPs). The CPP action is based mainly on the interaction between the positively charged (peptide) amino acids with the negatively charged moieties present at the surface of the biomembranes, including the BBB [223]. For example, the presence of the amino acids arginine and lysine facilitates the formation of hydrogen bonds with the negatively charged phosphates which are present on the (bio-)membranes. CPPs that showed different properties might undergo slightly different internalization mechanisms, including (specific and non-specific) endocytosis, pore formation and energy-dependent and -independent mechanism (via caveolin- and clathrin-independent lipid rafts) [132]. Among all CPPs, the modified HIV-1 transactivating transcriptional activator (TAT) peptide (having positive charges that can interact with negative charges of the BBB) has been successfully used for specific endocytosis delivery of liposome nanoparticles into the brain [224]. It has been demonstrated that nanoliposomes double-functionalized with a CUR derivative and with a TAT peptide enhances BBB crossing in vitro, carrying a CUR-derivative to bind Aβ peptide [225]. Also, CUR derivatives containing lipid ligands can be exploited for targeted drug delivery for brain diseases. Recently, the formation of nanoliposomes with CUR, or with CUR derivatives containing lipid ligands (phosphatidic acid, cardiolipin, or GM1 ganglioside) was able to inhibit the formation of fibrillar and/or oligomeric Aβ in vitro [226]. In Figure 7, we show a sketch of the two main mechanisms of liposome activity: direct penetration and the receptor-mediated transcytosis transport processes across the BBB.

Finally, multifunctional liposomes obtained by the suitable combination of more functionalities, that enhance both BBB transport and target diseased tissues, seem to be an efficient approach to design advanced treatments for AD and other brain diseases. Recently, multifunctional liposomes incorporating a lipid-PEG-CUR derivative and further functionalized with a BBB transport mediator (anti-transferrin antibody) evidenced an improved intake by the BBB cellular model [130].

### 5.3. Liquid Crystalline Nanocarriers

LCNs are self-assembled, thermodynamically stable, liquid crystalline nanostructures (e.g., bicontinuous cubic, inverted hexagonal or sponge phases) formed upon water dispersion of lyotropic lipids (such as unsaturated monoglycerides, phospholipids, glycolipids) and other amphiphilic molecules (surfactants). These amphiphilic molecules spontaneously self-assemble into organized nanostructures (such as cubosomes and hexosomes) containing hydrophilic and hydrophobic compartments which can encapsulate hydrophilic or lipophilic guest compounds [227]. Incorporation of drugs into LCNs for delivery processes through the BBB evidenced several advantages like controlled drug release, improved drug bioavailability, reduced chemical and physiological degradation, in vivo, and reduction of side effects [228,229]. Recently, entrapment of CUR into monoolein-based liquid crystalline nanoparticle dispersion (with almost 100% encapsulation efficiency) evidenced enhancement of the colloidal stability of CUR in the nanoformulation (about 75% of the CUR survived after 45 days of storage at 40 °C), while the in vitro release of CUR was sustained (10% or less over 15 days) [230]. Moreover, the release of CUR in bulk mesophases and in inverse hexagonal (HII) liquid crystals and the radical scavenging activity of LCNPs were also recently investigated [228]. The inverse hexagonal (HII) liquid crystals mesophases were constructed by a water solution of soybean lecithin (SL) and castor oil (Coil) and characterized by polarized light microscopy (POM), small-angle X-ray scattering (SAXS) and rheology. In that case, the biphasic drug sustained-release pattern for the LCNs evidenced a relatively fast release at the initial stage and then sustained release [228].

### 5.4. Macrocyclic Host-Macromolecules: Curcumin Loaded Cyclodextrin Nanocarriers

An efficient, alternative way to increase the water solubility of CUR consists in the complexation of CUR with macrocyclic host-macromolecules such as cyclodextrins. These macro- molecules have an interior hydrophobic surface which can host poorly water-soluble (macro-)molecules, while the external hydrophilic region ensures its aqueous solubility and colloidal stability [231]. They were proved to create CUR complexes and to improve its solubility [232,233,234]. The mechanisms for brain uptake and BBB crossing seems to be connected to a direct action of cyclodextrins that extract lipids (cholesterol and phospholipids) and some proteins from cell membranes (and lipid raft regions) modifying the molecular composition and properties of the lipid bilayers [231].

Recently, the therapeutic effect of CUR-cyclodextrinnanocarriers formulation on amyloid plaques in Alzheimer’s transgenic mice was demonstrated, in vivo, after intravenous and subcutaneous injection [233], and in vitro BBB model [234]. Alternatively, CUR complexation with calix(n)arenes macromolecules is also employed in brain drug delivery applications. Stable nanocarriers formed by combined methyl-β-cyclodextrin, *para*-sulphonato-calix(4)arene and *para*-sulphonato-calix(6)arene for the solubilization of CUR were recently investigated [232]. The nanocarriers, that self-assemble in a way that retains part of the CUR at the surface of the nanoparticles, showed a high affinity for the amyloid deposits, strongly labeling the SPs and also the diffuse deposits of AD brains. These nanocarriers were able to strongly label various amyloid aggregates in AD brains, thus proving their potential as trackers of AD pathology. Their biocompatibility was proved on several cell lines. Moreover, they were shown to interact with the Aβ peptide, reducing its aggregation and preventing the evolution of the disease and its toxicity [232]. We summarize in Table 1 the main characteristic and transport mechanism of the main CUR-conjugated nanocarriers.

### 5.5. Combining Therapeutic, Diagnostic and Stimuli-Responsive Functions: Theranostic Nanocarriers

Recently, the design of nanocarriers with combined therapeutic, diagnostic and stimuli-responsive multi-functionalities (theranosticnanocarriers) has stimulated research efforts concerning the treatment of different disease including brain disease and AD [235,236]. Different strategies can be used to engineer the surface of the nanoparticles, including the use of biomarkers, ligands, proteins and genes. Moreover, smart nanocarriers can take advantage of the specific microenvironment using (internal or external) stimuli-responsive triggers [237].

As an example, a CUR-conjugate, generation 3 PAMAM dendrimer (G3-Curc) nanocarrier proved to be a promising targeted theranosticnanocarrier for the treatment of glioblastoma brain tumors [238]. Together with the improvement of CUR water solubility and bioavailability, exvivo fluorescence imaging showed a tumor-specific distribution of G3-Curc conjugate (avoiding other major organs). While the *ex vivo* fluorescence imaging (and fluorescence microscopy) of the tumor tissue evidenced its specificity for nuclear distribution [238].

Moreover, a novel approach of CUR-conjugated superparamagnetic iron oxide (SPION) evidenced that amyloid plaque could be visualized in (ex vivo) magnetic resonance imaging (MRI) in AD mice, while no plaque was found in non-transgenic mice [144]. Significant accumulation and co-localization of amyloid plaques with nanoparticles were observed in an immune-histochemical analysis of the mouse brains. Therefore, this formulation has great potential for non-invasive diagnosis of AD using MRI. Furthermore, anti-amyloid antibody (IgG4.1)-conjugated gadolinium/magneticnanocarriers loaded with CUR/dexamethasone drugs were proposed for early diagnosis, targeting, and as a therapeutic agent(s) of cerebrovascular amyloid (CVA) [239]. The study evidenced that the nanocarriers efficiently distribute from the blood flow to the brain vasculature and target CVA deposit, owing to the IgG4.1. Thesetheranosticnanocarriers provide then both MRI and single-photon emission computed tomography (SPECT) agents contrast, specific to the CVA in the brain. In addition, they also carry CUR/dexamethasone therapeutic agents to reduce cerebrovascular inflammation associated with cerebral amyloid angiopathy (CAA), which is believed to trigger hemorrhage in CAA patients [239].

Finally, hybrid materials composed of mesoporous silica nanoparticles (MSNPs) have been, recently proposed as a promising class of versatile drug delivery nanocarriers, as well as an efficient nanoplatform for fluorescent cell tracking and bioimage applications [240,241,242,243]. The homogenous and regular nanostructure and the good biocompatibility of MSNPs facilitate the construction of an advanced biomedical platform for the delivery of therapeutics through the encapsulation of hydrophobic drugs inside the void volumes and the delivery of covalently linked therapeutic agents functionalized at the (large external/internal) silica surface [240,241,242,243]. Recently, CUR encapsulated MSNPs showed improved solubility, in vitro release profile and significantly enhanced cell cytotoxicity compared to the pure CUR [244]. MSNPs also provide a promising strategy to target cancer cells reducing peripheral nervous system uptake [245,246]. Recently, using a nano-templating approach, a novel MSNPs (with a size of ~220 nm) loaded with the CUR and chrysin have been developed for nose-to-brain delivery applications [247]. In that case, confocal microscopy experiments demonstrated that, following a 2 h incubation, the nanoparticles of <500 nm were able to accumulate within cells with fluorescein isothiocynate (FITC)-loaded MSNP showing membrane-localized and cytoplasmic accumulation [247]. These results evidenced the ability of the novel MSNPs to target and deliver active drugs into the CNS and bypass the BBB through olfactory drug delivery [247]. Moreover, a pH-responsive MSNPs (MCM-41) and capped by CS polymer (CS-MCM-41) were recently synthesized for the controlled CUR release near the cancer cells acidic environment [248]. The presence of CS acted as a pH-responsive shield to increase the solubility, bioavailability and anticancer efficacy of CUR against U87MG glioblastoma cancer cell line. Cytotoxicity investigations on the U87MG glioblastoma cancer cell line evidenced, in fact, that CS-MCM-41 nanoparticles have more cytotoxicity than free CUR and CUR-loaded MSNPs (MCM-41) without CS [248].

In conclusion, the suitable combination of those multi-functional properties allows identifying, within a single theranostic nano-platform, the diseased tissue location, the nanocarrier delivery, and the biodistribution, thus allowing profitable monitoring of the progress/efficacy of the therapeutic treatment. The targeting of overexpressed receptors on brain diseased tissues and cells allows a specific release of CUR cargo in the target site [235]. Moreover, real-time monitoring of the pharmacokinetic profiles and target site accumulation may direct the proper selection of treatment and therapy. Finally, the improvement of theranostic approaches and the assessment of the therapeutic efficacy may stimulate the development of personalized medicine-based therapeutic protocols of interventions.

## 6. Conclusions and Future Remarks

AD and other brain diseases are an important cause of human deaths worldwide. New alternative therapies for AD and neurodegenerative diseases arise from ongoing research in the whole world. Because CUR may cross the hematoencephalic barrier, studies have shown that it leads to various improvements in the pathological process of AD. The molecular mechanisms of CUR in AD are several, including antioxidant, Aβ-binding, anti-inflammatory, tau inhibition, metal chelation, neurogenesis, and synaptogenesis promotion. These effects are scientifically verified, with CUR revealing an outstanding performanceon prevention, treatment and diagnosis of AD. However, in this success story of CUR, there is the fact that its bioavailability is too lower. To remove this problem, new formulations such as nanoCUR are developed. CUR nanoformulations are a therapeutic alternative in a new discovery phase, being nontoxic for other body cells.

Regarding nanocarriers formulation for CUR delivery, SLNs, NLCs, liposomes, LCNs and macrocyclic host macromolecules reported interesting characteristics that need to be more studied to a better to understand their mechanism of action and effectiveness. Future studies need to test this CUR nanoformulation, and different combinations and formulation, in brain diseases. New efforts are needed to test new CUR nanomedicine formulations, with better CUR bioavailability, in brain diseases.

## Figures and Tables

**Table 1:** Characteristic and the transport mechanism of the main curcumin-conjugated nanocarriers.

Nanocarrier Type	Most Investigated Components	Shape/Size	Advantages/Disadvantages	Mechanisms for Brain Uptake and BBB Crossing
Polymer Nanoparticles 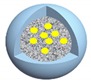	Poly(lactic-co-glycolic acid) (PLGA) is the most investigated polymer. Poly(butyl)cyanoacrylate (PBCA) and Chitosan (CS) also investigated.	Globular (10–200 nm)	Tunable physicochemical properties (through the choice of component polymers), easy preparation method, controlled pharmacokinetic, high biocompatibility, biodegradability, neurotoxic	Endocytosis and/or transcytosis through the endothelial cells, tight junctions opening. Surface conjugation with targeting ligands improve the transcytosis across the BBB [178,182,188].
Micelles 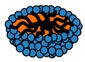	PLGA-PEG diblock and PLGA-PEG-PLGA triblock copolymers	Spherical (20–100 nm)	Negligible neurotoxic effects, improved drug bioavailability, high physicochemical and colloidal stability, sustained and controlled release/can be used only for lipophilic (hydrophobic) drug, slow drug loading capacity.	Endocytosis and/or transcytosis. Surface conjugation with targeting ligands improve the transcytosis across the BBB [198,199]
Solid Lipid Nanoparticles 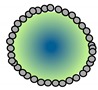	Glyceride derivatives (complex glyceride mixtures, triglycerides, monoglycerides, hard fats, stearic acid, cetyl alcohol, cholesterol butyrate, emulsifying wax. The lipid core is usually stabilized by surfactants (about 1–5% w/v) and/or cosurfactant (such as poloxamer 188 and/or Tween^®^ 80)	Spherical (50–300)	High entrapment efficiency for hydrophobic drugs, biocompatibility, high physical stability and drug protection, controlled release, ease of formation methods (that can be easily scaled up and do not require organic solvents thus avoiding (neuro-)toxicity)/reduced hydrophilic drug entrapment efficiency, sterilization difficulties	Brain uptake by the paracellular pathway through the opening of the tight junctions in brain microvasculature, passive diffusion, and endocytosis. Active targeting with receptors (apolipoprotein E) [204,206,210]
Liposome 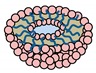	Lipids:1,2-dipalmitoyl-sn-glycero-3-phospho-choline ethyl-phosphatidyl-choline (DPPC), phosphatidylcholine (PC), sphingomyelin (SP), and lecithin (LC),Cholesterol. PEGylated 1,2-distearoyl-sn-glycero-3-phospho-ethanolamine-PEG 2000 (DSPE)	Globular/lamellar (20–200 nm)	Possibility of entrapping both hydrophilic and hydrophobic drugs, high drug protection and targeting efficiency/neurotoxicity, physicochemical instability, the tendency of fusion, rapid clearance, sterilization difficulties	Passive targeting, adsorption-mediated transcytosis, or receptor-mediated endocytosis. Active targeting. with receptors glutathione, glucose, transferrin, lactoferrin, apolipoprotein E, phosphatidic acid. Use of cell-penetrating peptides (CPPs - such as TAT, penetratin) [215,216,218,219]
Liquid Crystalline Nanocarriers 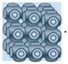	Unsaturated monoglycerides, phospholipids, glycolipids and surfactants	Bicontinuous cubic (cubosome), inverted hexagonal (hexosomes) or sponge phases (20–200 nm)	Enhancement colloidal stability, controlled andsustained (in vitro) release of curcumin, improved drug bioavailability, reduced chemical and physiological degradation (in vivo), reduction of side effects	Passive targeting, adsorption-mediated transcytosis, or receptor-mediated endocytosis. [228,229]
Cyclodextrins 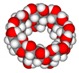	Mainly the β-cyclodextrin derivatives	Cyclic (150–500 nm)	High biocompatibility, lipophilic cavity sensitively improve curcumin solubilization, outer hydrophilic surfacefacilitate dispersion and colloidal stability of the formulation	The direct action of cyclodextrin by extracting lipids (cholesterol and phospholipids) and some proteins from cell membranes (and lipid raft region) modifying the molecular composition and properties of the lipid bilayers. [231,232,233,234].
